# A genomic copy number variant analysis implicates the *MBD5* and *HNRNPU* genes in Chinese children with infantile spasms and expands the clinical spectrum of 2q23.1 deletion

**DOI:** 10.1186/1471-2350-15-62

**Published:** 2014-05-29

**Authors:** Xiaonan Du, Yu An, Lifei Yu, Renchao Liu, Yanrong Qin, Xiaohong Guo, Daokan Sun, Shuizhen Zhou, Bailin Wu, Yong-hui Jiang, Yi Wang

**Affiliations:** 1 Division of Neurology, Children’s Hospital of Fudan University, 399 Wan Yuan Road, Shanghai 201102, China; 2 Institute of Biomedical Sciences and MOE Key Laboratory of Contemporary Anthropology, Fudan University, Shanghai 200032, China; 3 Boston Children’s Hospital, Harvard Medical School, Boston, MA, USA; 4 Division of Medical Genetics, Department of Pediatrics and Neurobiology, Duke University School of Medicine, 905 S. LaSalle ST, Durham NC 27710, USA

**Keywords:** Infantile spasms, Copy number variants, Array CGH, Autism spectrum disorders, *MBD5*, *HNRNPU*

## Abstract

**Background:**

Infantile spasms (IS) is a specific type of epileptic encephalopathy associated with severe developmental disabilities. Genetic factors are strongly implicated in IS, however, the exact genetic defects remain unknown in the majority of cases. Rare mutations in a single gene or in copy number variants (CNVs) have been implicated in IS of children in Western countries. The objective of this study was to dissect the role of copy number variations in Chinese children with infantile spasms.

**Methods:**

We used the Agilent Human Genome CGH microarray 180 K for genome-wide detection of CNVs. Real-time qPCR was used to validate the CNVs. We performed genomic and medical annotations for individual CNVs to determine the pathogenicity of CNVs related to IS.

**Results:**

We report herein the first genome-wide CNV analysis in children with IS, detecting a total of 14 CNVs in a cohort of 47 Chinese children with IS. Four CNVs (4/47 = 8.5%) (1q21.1 gain; 1q44, 2q31.1, and 17p13 loss) are considered to be pathogenic. The CNV loss at 17p13.3 contains *PAFAH1B1* (*LIS1*), a causative gene for lissencephaly. Although the CNVs at 1q21.1, 1q44, and 2q23.1 have been previously implicated in a wide spectrum of clinical features including autism spectrum disorders (ASD) and generalized seizure, our study is the first report identifying them in individuals with a primary diagnosis of IS. The CNV loss in the 1q44 region contains *HNRNPU*, a strong candidate gene recently suggested in IS by the whole exome sequencing of children with IS. The CNV loss at 2q23.1 includes *MBD5*, a methyl-DNA binding protein that is a causative gene of ASD and a candidate gene for epileptic encephalopathy. We also report a distinct clinical presentation of IS, microcephaly, intellectual disability, and absent hallux in a case with the 2q23.1 deletion.

**Conclusion:**

Our findings strongly support the role of CNVs in infantile spasms and expand the clinical spectrum associate with 2q23.1 deletion. In particular, our study implicates the *HNRNPU* and *MBD5* genes in Chinese children with IS. Our study also supports that the molecular mechanisms of infantile spasms appear conserved among different ethnic backgrounds.

## Background

Infantile spasms(IS; also known as West syndrome) is a severe and specific type of epilepsy syndromes or epileptic encephalopathies that presents in early infancy. IS is characterized by the early onset (4–8 months of age) of the epileptic form of spasm, a distinct hypsarrhythmia on EEG, and poor developmental outcome [1]. The incidence of IS ranges from 0.25 to 0.4 per 1000 living births in Western countries. The prevalence of IS in Chinese children is not known but is estimated to be similar to that observed in the Caucasian population. Due to its refractory nature to most antiepileptic treatments and its frequent association with poor cognitive development, IS is one of the most devastating epileptic encephalopathies and neurological disorders of infancy and early childhood. Interestingly, IS has been shown to be strongly associated with autism spectrum disorders (ASD) [2]. Infantile spasms is a prominent clinical feature in several high profile monogenic syndromes such as tuberous sclerosis complex (TSC). Mutations in a growing number of genes have also been reported in individuals with nonsyndromic infantile spasms by medical re-sequencing or whole exome sequencing projects [3-5]. These genes include *ARX, CDKL5, FOXG1, GRIN1, GRIN2A, MAGI2, MEF2C, SLC25A22, SPTAN1*, *CACNA1A, CHD2, FLNA, NEEDL4*, and *STXBP1*. Despite these findings, the causes for the majority of infantile spasms cases remain unknown. Recently, rare and recurrent genomic copy number variants (CNVs) have been implicated in intellectual disabilities, ASD, and other neuropsychiatric disorders. Many of these same CNVs have also been implicated in individuals with generalized epilepsy in Caucasian population [6-11]. However, the role of CNVs in IS has not been fully investigated. The clinical presentations of IS in Chinese children remain poorly characterized. There are few published reports on genomic or genetics studies of IS in Chinese children. We hypothesized that CNVs also contribute significantly to the susceptibility of IS in Chinese children. We conducted the first genome-wide copy number variation analysis in 47 Chinese children with IS using an array comparative genomic hybridization (array CGH) technique. We detected a total of 364 CNVs by array-CGH and validated 14 rare, inherited or *de novo* CNVs by further molecular experiments in this cohort. Genomic and medical annotations provided evidence that support the pathogenicity of several new CNVs, specifically, those in the *HNRNPU* and *MBD5* genes, in IS.

## Methods

### Study subjects

The study was approved by the institutional review board (IRB) of the Children′s Hospital of Fudan University. All the photographs of the children with or without eyes blocked have consent from their parents. All samples and information were collected after informed consent was obtained from the parents. The diagnosis of infantile spasms was made based on an assessment of clinical seizure presentation and electroencephalography (EEG) recorded by a pediatric neurologist with experience in the clinical diagnosis of infantile spasms (Additional file 1: Table S1). All patients included in this study had cranial magnetic resonance imaging (MRI), G-band karyotype and basic metabolic screening tests. The detailed medical findings and family history were also obtained. All patients with a documented history of infection in the central nervous system, a significant history of hypoglycemia, hypoxic ischemic encephalopathy, extreme premature birth, or other documented non-genetic insults were excluded. In addition, any cases with a clinical diagnosis of neurocutaneous syndromes such as tuberous sclerosis complex or other known genetic syndromes were not enrolled in this study.

### Comparative genomic hybridization or microarray analysis

The array CGH was performed using an Agilent Human Genome CGH microarray 180 K kit (Agilent Technologies Inc. Shanghai, China) with a resolution of 6.4 kb. For each sample, 1.5 μg of genomic DNA was used for each array CGH experiment. The array CGH experiments were performed according to the manufacturer’s instruction as described previously [12]. Briefly, genomic DNA from patients and controls was digested with the restriction enzymes A*lu*I and *Rsa*I (Promega) and fluorescently labeled with Cy5 (patients) or Cy3 (controls) using the Agilent DNA Labeling Kit. Labeled DNAs were combined, denatured, pre-annealed with Cot- DNA (Invitrogen) and blocking reagent (Agilent), and then hybridized to the arrays for 40 hours in a rotating oven (Agilent Technologies) at 65°C and a speed of 20 rpm. After performing the hybridization step and the recommended washes, the arrays were scanned at 5 μm resolution with an Agilent G2505A scanner. The images were analyzed using the Feature Extraction software (Agilent Technologies, Shanghai, China). All array data that passed the quality metrics were further processed by the DNA Analytics software.

### Array CGH data analysis and CNV calling

Array CGH data were analyzed using the Cytogenomics v2.5 software (Agilent Technologies, CA,USA). The QC metrics table was used to check the signal intensities and background noise. A DLR (Derivative Log Ratio) score above 0.20 was set as the cutoff criteria and those with below DLR 0.20 score indicate poor quality of array data and possibility of false CNV calling. CNV calls were performed using the ADM2 algorithm with a sensitivity threshold of 6.0 and a minimum of 5 probes. The non-benign CNVs were identified using common CNV filter including Database of Genomic Variants (DGV) and published CNV dataset from Chinese Han individuals.

### Validation of CNVs by quantitative real-time PCR (qPCR)

For each CNV identified in this study, we confirmed the CNV gain or loss using quantitative PCR (qPCR) in probands and their parents. The primers used for qPCR were derived from the candidate genes within the CNVs that were also related to genetic disease in the OMIM database. Three to five primer pairs for each gene were selected for quantitative PCR (qPCR). The sequence of these primer pairs are listed in Additional file 1: Table S2. PCR reactions were performed in a volume of 10 μl containing 5 μl SYBR Green I Master mix (Toyota, China), 0.4 mM primers, and approximately 25 ng template cDNA. The housekeeping gene HMBS was used as an endogenous control for normalization. The reactions for each sample were performed in triplicate using a StepOnePlus™ Real-Time PCR System (Applied Biosystems®). The thermal profile for the qPCR was as follows: a pre-incubation step of 45 sec at 95°C, followed by 40 cycles of 5 sec at 95°C, 30 sec at 60°C, and 30 sec at 72°C. The raw data were analyzed using StepOne™ Software v2.1. Amplification levels were calculated using the ∆∆Ct method according to the manufacturer’s instructions.

### Sequence analysis of *MBD5* and *ORC4*

Primers flanking the *MBD5* (NM_018328) and *ORC4* (NC_000002) exon-intron junctions were designed to amplify the exons of these two genes using the Primer 3 online program. The sequences of these primers are listed in the Additional file 1: Table S3. Touchdown PCR was performed for all of the other amplicons. Briefly, cycling conditions comprised 10 cycles of: 30 sec denaturation at 95°C, 30 sec annealing and 72°C, 40 sec for primer extension. The annealing temperature was decreased 0.5°C every second cycle from 63°C to 58°C, followed by 25 additional cycles with an annealing temperature of 58°C. The direct sequencing of PCR products was carried out using an ABI Prism Big Dye System (Applied Biosystems). The raw sequencing data were processed, and variant calling was performed using the Mutation Surveyor v3.97 software program (SoftGenetics, PA, USA).

### Statistical analysis

Data were analyzed using SPSS (Statistical Package for the Social Sciences) version 18.0 statistical software (SPSS Inc., Chicago, IL). A one-way ANOVA test followed by LSD was used when comparing three groups. A p value of p < 0.05 (two-tailed) was considered significant.

## Results

### Study subjects

A total of 47 patients (24 males and 23 females) with a clinical diagnosis of IS were enrolled in this study (Additional file 1: Table S1). The diagnosis of IS was supported by both a characteristic clinical presentation of seizures and an abnormal EEG with hypsarrhythmia that is unique to IS in 45 patients. Two patients exhibited clinical presentations of infantile spasms without a typical EEG pattern of hypsarrhythmia in the recording at the time of enrollment (aged 8 months-old and 13 months-old). The mean age of subjects at enrollment was 20.2 ± 19.7 months. The average onset of infantile spasms was 6.3 ± 2.9 months.

All patients displayed developmental delay and intellectual disability. Brain structural MRI analyses were performed for all participants. Abnormalities including pachygyria, corpus callosum dysplasia, enlarged CSF space, diffuse and abnormal cortical migration, and atrophy were found in 5 patients (Additional file 1: Table S1). Other congenital anomalies, including heart defects and limb anomalies, were found in 7 out of 47 cases. The parents of all the probands were reported to be healthy and did not have history of seizure disorders. One family (S133) had two affected boys (2 and 8 years-old) with IS, motor development delay, speech and cognitive impairment.

### Genome-wide copy number variation analysis

G-band karyotyping and routine clinical biochemical screening tests for inborn errors of metabolism were performed in all participants to rule out common known genetic and metabolic conditions. No specific abnormalities were identified in these tests. An array CGH analysis was performed for all probands using an Agilent Human Genome CGH microarray 180 K array. The CNV calling and filtering were described previously [12]. A Han Chinese specific CNV dataset obtained from multiple sources was used for the CNV analyses [13-15]. The CNVs called by the computational program were validated primarily by real-time quantitative PCR (qPCR) analyses. A total of 364 CNVs were identified from 47 individuals (7.7 CNVs/per case). After filtering and annotation, 14 CNVs (5 deletions and 9 duplications) were further validated by molecular experiment by the real time qPCR using the primers within the genes mapped to the deleted or duplicated intervals (Additional file 1: Table S2). The sizes of the CNVs ranged from 115 kb to 4.1 Mb. One proband (S34) had two CNVs while the rest of the cases had a single CNV. Parental testing was performed on both parents for 12 CNVs. Six CNVs were *de novo* and 6 were either maternally or paternally inherited. In other two families (S2 and S75), parental test was not performed because either one or both parents were not available for testing.

We conducted genomic and medical annotations for each CNV that were identified and confirmed from this cohort. The genomic annotation was performed by reviewing the function of the candidate genes residing within the deleted or duplicated interval of the CNVs and absence in the DGV. The medical annotation was performed by reviewing the clinical reports related to each of the CNVs in PubMed, the candidate gene(s) in CNVs relevant to the OMIM entries, and the function of these genes in the evidence provided by other model organisms or experimental systems. Through these analyses, we determined the pathogenicity of the CNVs in IS based on a comprehensive assessment of the existing clinical data, the size of the CNV, the gene content of the CNV, and the inheritance of the CNV. These CNVs were placed into 3 classes based on the evidence supporting their pathogenicity in IS: 1) pathogenic; 3) unknown clinical significance; and 3) probably benign (Table 1). The molecular and clinical characteristics of each class of CNVs are discussed below.

**Table 1:** Rare and novel CNVs identified from Chinese infantile spasms children

**Subject**	**Sex**	**Onset of IS (month)**	**EEG (HYPS+/-)**	**Genomic location/coordinates (hg19)**	**Genomic event**	**Size**	**Inheritance**	**Candidate gene(s)**
**Pathogenic**								
S100	M	4	+	chr17:2,405,454-2,520,464	Del	115 Kb	De novo	*PAFAH1B1*
S37	M	5	+	chr1:244,961,797-247,074,490	Del	2.1 Mb	De novo	*HNRNPU*
S162	F	6	-	chr2:147,953,313-152,061,251	Del	4.1 Mb	De novo	*MBD5*
S67	F	10	+	chr1:145,764,453-147,824,207	Dup	2.0 Mb	De novo	*CHD1L*
**Unknown clinical significance**								
S15	F	2	+	chr10:84,222,075-85,293,140	Dup	1.0 Mb	Paternal	*NRG3*
S34	M	5	+	chrX:102,262,951-102,659,333	Dup	396 kb	Maternal	*NGFRAP1*
S134	F	4	+	chr17:14,111,754-15,442,119	Del	1.3 Mb	Paternal	*PMP22*
S163	F	4	+	chrX:6,451,691-8,115,193	Dup	1.6 Mb	Paternal	*STS,VCX3A*
S165	M	5	+	chr11:101,857,720-102,256,635	Del	398 kb	De novo	*YAP1*
**Probably benign**								
S2	M	3	+	chr2:216,898,976-217,160,487	Dup	261 kb	Not determined	
S34	M	5	+	chr2:137,826,656-138,048,583	Dup	221 kb	Paternal	
S42	M	4	+	chr4:48,983,002-49,063,489	Dup	80 kb	Paternal	
S75	M	6	+	chr6:118,989,529-120,238,639	Dup	1.2 Mb	Not determined	
S133	M	13	-	chr8:135,644,952-135,791,363	Dup	146 Kb	De novo	

### Pathogenic CNVs

**Figure 1:**
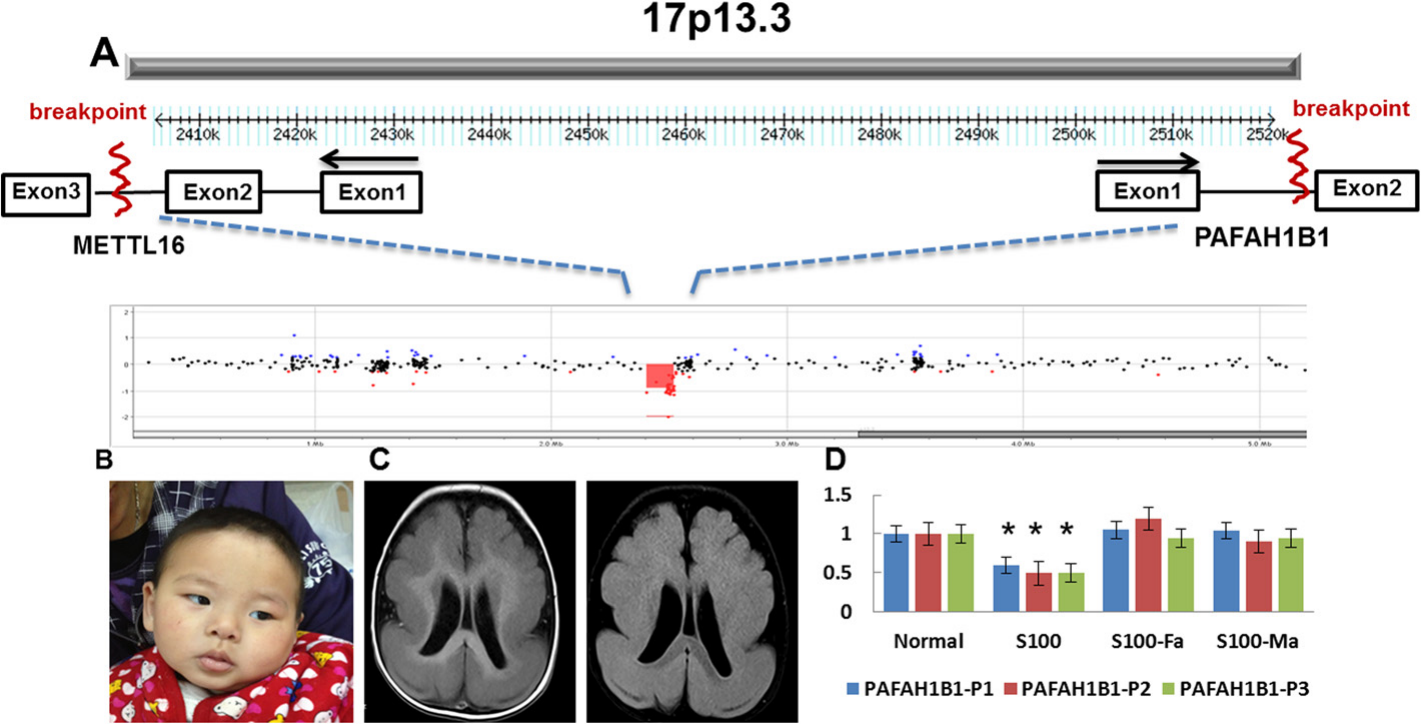
**A patient (S100) with a 115-kb microdeletion at 17p13.3 that disrupts the *PAFAH1B1 *(*LIS1*) gene. A)** A local view of the 17p13.3 deletion. The proximal breakpoint is within intron 2 of *METTL16*, a gene that encodes methyltransferase-like 16 protein whose exact function has not been characterized. The distal breakpoint is within intron 1 of *PAFAH1B1* (*LIS1*), a known causative gene for lissencephaly. The black arrows are the direction of transcription. **B)** The clinical features of a patient with a prominent forehead and micrognathia. **C)** Structural MRI analysis showed grade III lissencephaly. **D)** Analyses by qPCR confirmed that the copy number loss in this patient was *de novo* using three different primers (P1-P3) within the *PAFAH1B1* and analyses of both parents. *p < 0.01, proband vs parents and control.

Four CNVs were classified as pathogenic (Table 1). In case S100, a *de novo* 115-kb deletion was detected in the 17p13.3 region (chr17:2,405,454-2,520,464, http://www.genome.ucsc.edu; hg19, thereafter) in a boy diagnosed with IS (Figure 1A and D). The proximal breakpoint of the deletion is within intron 2 of *METTL16*, a gene that encodes a methyltransferase-like protein. But the exact function of this protein has not been determined biochemically. The distal breakpoint was within an intron 1 of *PAFAH1B1* (*LIS1*), a gene known to cause lissencephaly and the feature of IS [16]. This patient developed IS at 4 months of age and the diagnosis of IS was supported by an EEG pattern of hypsarrhythmia. The patient had mild dysmorphic facial features including hypertelorism and micrognathia (Figure 1B). Structural brain MRI analysis revealed diffuse and generalized pachygyria and grade III to IV lissencephaly (Figure 1C) that is consistent with the clinical findings in other patients with similar defects of *PAFAH1B1*. Generalized seizures and infantile spasms are common clinical features associated with deficiency of *PAFAH1B1*[16,17].

**Figure 2:**
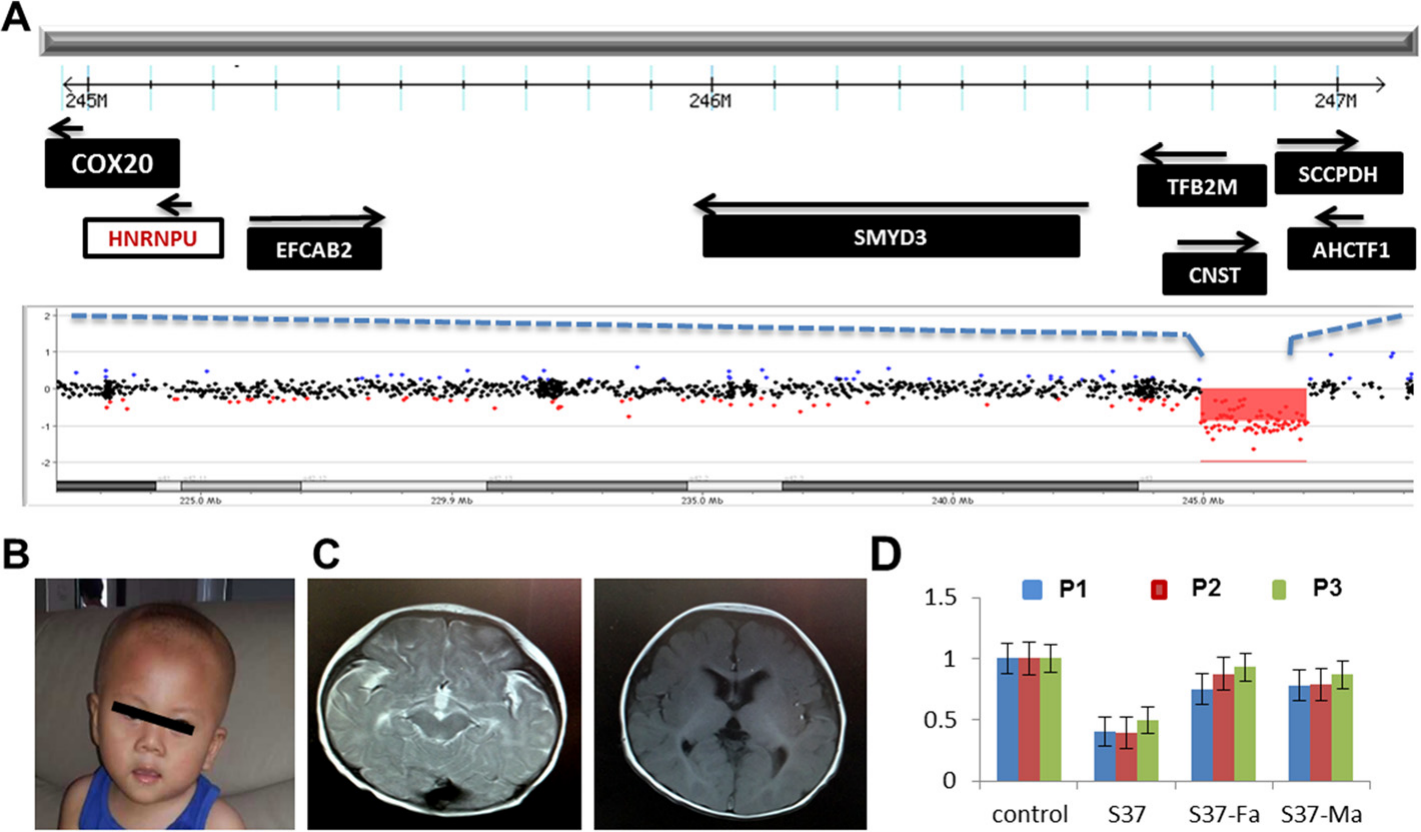
**The proband of S37 with a 2.1-Mb microdeletion at 1q44 that disrupts the infantile spasms candidate gene *HNRNPU *reported by the WES study. A)** A local view of the 1q44 deletion and genes in the deleted interval. The black arrows are the direction of transcription of genes. **B)** The clinical features of a proband with a dysmorphic face. **C)** Structural MRI analysis showed delayed myelination in the brain. **D)** qPCR confirmed the copy number loss in probands using three different primers (P1-P3) within *HNRNPU*. *p < 0.01, proband vs parents and control.

In case S37, a *de novo* 2.1-Mb deletion was found in the 1q44 region (chr1:244,961,797–247,074,490) in a boy with a primary diagnosis of IS (Figure 2A and D). The patient developed IS at 5 months of age and the EEG indicated hypsarrhythmia and epileptiform of activity. Physically, he had a prominent forehead and small chin (Figure 2B). Structural brain MRI analysis showed delayed myelination and some degree of cortical dysplasia (Figure 2C). There was a history of developmental regression after the onset of IS. At 2 years of age, his language development was severely delayed. His behavioral profile was consistent with a clinical diagnosis of ASD. The deleted region contains 8 known genes including *COX20, HNRNPU, EFCAB2, SMYD3, TFB2M, CNST, SCCPDH*, and *AHCTF1* (Figure 2A). Interestingly, a sequence variant at the splice acceptor site of *HNRNPU* was found in a patient with infantile spasms by whole exome sequencing (WES) in Epi4K project [14,18]. Our findings thus provide further support that *HNRNPU* in the 1q44 region is pathogenic or causative gene for infantile spasms in this case.

**Figure 3:**
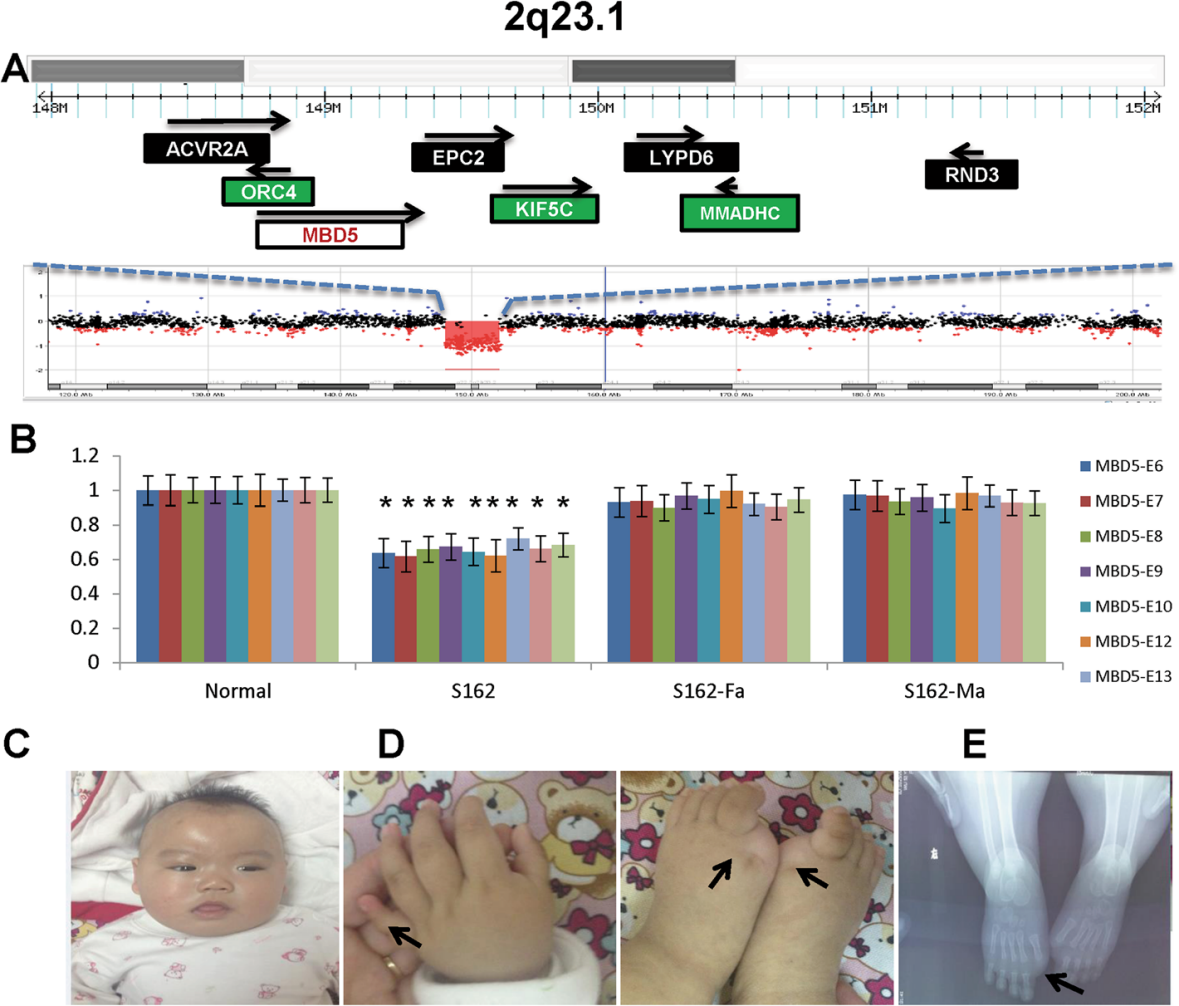
**A patient from S162 with a 4.1-Mb microdeletion at 2q23.1 that disrupts the known ASD causative gene *MBD5*. A)** A local view of the 4.1 Mb 2q31.1 deletion and genes in the deleted interval. **B)** Analysis by qPCR confirmed the copy number loss and de novo event in the patient using 7 primers (MBD5E6-E13) from exons 5–13 of *MBD5*. **C)** The clinical features of a proband with a dysmorphic face, including a coarse face. **D)** Clinodactyly of the fifth finger, excessive fat pads of the second and middle fingers, and the absence of great toes. **E)** X-ray analyses showed the absence of the distal and proximal phalanges of the great toes. *p < 0.01, proband vs parents and control.

We also detected a deletion in the 2q23.1 region including *MBD5* gene in case S162 (Table 1 and Figure 3A) and a duplication in the 1q21.1 region in case S67 (data not shown), respectively. The CNVs in these two regions have previously been implicated in a wide spectrum of clinical presentations that include ASD, intellectual disability and seizure, but not specifically IS [19-21]. In case S162, a 4.1-Mb deletion in the 2q23.1 region was found in a 7 month-old female (Figure 3A and B). The size of the deletion was moderate compared to other cases in the literature that ranged from 100 kb to 6 MB in the region [20]. The seizures in proband started at 4 days of age and then transformed into spasms at 6 months of age. An EEG study indicated the presence of a bilateral sharp wave and spike slow wave complex at 8 months of age that was suggestive of hypsarrhythmia. Structural brain MRI analysis showed some degree of cortical dysplasia and microcephaly. She also had dysmorphic face including a broad forehead, arched eyebrows, and deep nasal bridge (Figure 3C). Her great toes were bilaterally absent (absent hallux) and the fifth fingers were short and exhibited clinodactyly (Figure 3D). An X-ray study revealed missing proximal and distal phalanges in the great toes but normal knee joints. She also had significant hypotonia, severe language delay, and suspected ASD diagnosis.

The 2q23.1 deletion has been reported in individuals with ASD, seizure, and developmental delay. However, the overall neurological presentations in our case appeared much more severe than in other individuals with similar sized deletions in 2q23.1 in three other major reports considering the degrees of hypotonia and the onset of seizures [20,22-24] (Table 2). Although the mild anomalies of hand and foot has been seen in most patients with *MBD5* defect, the features of IS and the absent hallux limb anomaly have not been reported previously. We then tested the hypothesis that the sequence variants for the genes in the deleted interval of the non-deleted chromosome may modify or contribute to more severe and unique clinical presentations in this case. There are a total of 8 genes within the deleted intervals (chr2:147,953,313-152,061,251) in our case (Figure 3A), including three classified as known disease-causing genes listed in the OMIM: *MBD5*, *ORC4*, and *MMADHC. MBD5* is an important gene within the 2q23.1 region that was believed to be responsible for ASD, intellectual disabilities, developmental delays and seizures in 2q23.1 deletion because point mutations in *MBD5* shared the key neurological features with 2q23.1 deletion (Tab1e 2) [20,22-24]. *ORC4* is a causative gene of a subtype of Meier-Gorlin syndrome (MGS), a rare autosomal recessive disorder characterized by severe intrauterine and postnatal growth retardation, developmental delay, microcephaly, bilateral microtia, and aplasia or hypoplasia of the patellae. In our case, the X-ray study did not reveal abnormal patellae. The clinical presentations are not consistent with the diagnosis of MGS. The *MMADHC* gene is responsible for the cobalmin (cbl)-D type of methylmalonic aciduria. The metabolic profile in our case did not suggest a defect in cobalmin metabolism. Because the functions of both *MBD5* and *ORC4* are important for brain function, we tested whether sequence variants of *MBD5* and *ORC4* in the non-deleted allele were present in this patient. We sequenced the coding exons of *MBD5* and *ORC4*. The primers used for sequencing and PCR are included in Additional file 1: Table S3. No significant sequence variants were identified in all coding exons of *MBD5* and *ORC4* genes.

**Table 2:** **Comparison of clinical features of 2q23.1 deletion in this study and other cohorts or** ***MBD5*** **specific mutation**

**Clinical Features**	**MBD5-specific mutation**^**20,22–24**^		**2q23.1 Deletion**^**20,22–24**^		**Case in this study**
	**Number**	**Percentage**	**Number**	**Percentage**	**+, present; - absent**
**Neurological**					
Development delay	20/21	95%	53/55	96%	+
Language impairment	12/21	57%	38/55	69%	+
Seizure	11/21	52%	32/55	58%	+
Infantile spasms	0/21	0	0/55	0	+
Infantile hypotonia	4/21	19%	23/55	41%	+
**Growth/Endocrine**					
Short stature	5/21	24%	25/55	45%	-
Local hirsutism	0/21	0	4/55	7%	-
**Craniofacial abnormalities**					
Coarse face	0/21	0	3/55	5%	+
Broad forehead	3/21	14%	4/55	7%	+
Microcephaly	1/21	5%	26/55	47%	+(<2SD)
Synophrys	2/21	9%	10/55	18%	+mild
Nasal abnormalities	7/21	33%	35/55	64%	+
Open mouth	3/21	14%	23/55	42%	+
Downturned corners of the mouth	3/21	14%	17/55	31%	+
Macroglossia or protruding tongue	2/21	9%	6/55	11%	-
Dental abnormalities	4/21	19%	13/55	24%	
**Skeletal abnormalities**					+
Small hands and feet	1/21	5%	23/55	42%	+
Clinodactyly, 5th finger	2/21	9%	22/55	40%	+
Brachydactyly	0/21	0	13/55	24%	+
Short fifth digit of hands/feet	1/21	5%	14/55	25%	+
Absent hallux	0/21	0	0/55	0	+
**Behaviors**					-
Autistic feature	13/21	62%	44/55	80%	+
Sleep disturbance	5/21	24%	17/55	31%	ND
Self-injurious behavior	2/21	9%	17/55	31%	+
**Other**					-
Cardiovascular abnormalities	1/21	5%	4/55	7%	-
Urogenital abnormalities	1/21	45%	1/55	2%	-

ND, not documented.

In case S67, a *de novo* 2 Mb duplication at 1q21.1 (chr1:145,764,453- 147,824,207) was found in a 4-year-old girl with a diagnosis of IS. The duplication spanned the 1q21.1 distal region but did not include the TAR (thrombocytopenia with absent radius) syndrome region. The size of this duplication is larger than many of the cases described by Brunetti et al. [25] and Mefford et al. [26] but is similar to other recently reported cases [27]. The probe coverage may contribute to the discrepancy for the estimated size of duplication in different reports. Duplication at 1q21.1 has been implicated in a wide spectrum clinical presentations including ASD, congenital heart defects, seizures, schizophrenia, and developmental delay [25-27]. In our case, the proband developed IS at 8 months of age with EEG of hypsarrhythmia epileptiform activity. The spasms were poorly controlled and progressed to Lennox-Gastaut syndrome at a later age. Structural brain MRI analysis uncovered mild delayed myelination and enlarged ventricles. Her cognitive development regressed significantly after the onset of spasms. There are more than 20 genes within the 2 Mb duplicated interval and the knowledge for which gene or genes are responsible for neurological phenotypes is limited. *CHD1L*, a gene encoding chromodomain helicase DNA binding protein 1-like, was mapped to this interval.

### CNVs of unknown clinical significance

Five novel *de novo* or inherited CNVs were considered to have potential roles in IS because of their size and the known function of the gene in the brain. However, the exact clinical relevance could not be determined with confidence. These CNVs include the following: 1) a paternally inherited 1.07 Mb duplication in chromosome 10q22.3 that contains *NRG3*, a gene implicated in schizophrenia and other neuropsychiatric diseases [28,29], 2) a maternally inherited 396 kb duplication at Xq22.1 (chrX:102,262,951-102,659,333) in a boy (S34). The duplication contains *NGFRAP1* (*NADE*), a gene that encodes a nerve growth factor receptor-associated protein. The NGFRAP1 protein notably interacts with TSC1 [30], a protein responsible for TSC, a genetic disorder in which infantile spasms is a common feature; 3) a pateral1y inherited 1.6 Mb duplication at Xp22.31 in S163 that contains two OMIM genes of *STS* and *VCX3A* (chrX:6,451,691- 8,115,193). The deletion including *STS* and *VCX3A* was found in two cases with ichthyosis and/or mental retardation. The duplication of Xp22.31 region has been reported but the clinical relevance has not been determined [31,32]. The function of *STS* and *VCX3A* in brain is not known; 4) a paternally inherited deletion in the 17p12 region that contains *PMP22*, a gene that causes a hereditary neuropathy with liability to pressure palsies (HNPP, OMIM 162500) [33]; and 5) a *de novo* 398-kb deletion in the 11q22.1 region in S165 (chr11:101,857,720-102,256,635) that contains a gene encoding YAP1, a protein that associates with 14-3-3 in an AKT-dependent manner that is important for brain function [34].

Although these CNVs are rare and novel, their roles in the clinical presentation of IS are not immediately clear. In the case with the CNV gain at 10q23.1, the *Neuregulin 3* (*NRG3)* gene, a family of neurally expressed proteins that perform a wide range of functions in the developing nervous system, mapped within this region. The breakpoint of duplication was within the middle of *NRG3* coding exons. Therefore, it is not clear what was the impact of this duplication to the expression of *NRG3.* There are numerous reports suggesting that *NRG3* contributes to the susceptibility of schizophrenia and other neuropsychiatric disorders [28,35,36]. In our case, because the duplication is inherited from the healthy parent, the role of this duplication in proband related to IS could not be determined with confidence. In the case S34, a 396 kb duplication in X chromosome containing *NGFRAP1(NADE)* gene in boy was inherited from the healthy mother. The similar duplication has not been reported before associated with disease in humans. The function of *NGFRAP1* related to *TSC1* suggested that the *NGFRAP1* may be a risk factor for the IS. The similar scenario is applied to the case of S165 with a *de novo* 398-kb deletion in the 11q22.1 region. YAP1, a protein important for brain function is mapped to the region but the similar deletion has not been associated with disease phenotype in human. In case S134, the father carrying the same deletion containing *PMP22* gene appears healthy and does not have clinical symptoms, suggesting a diagnosis of PMP22-related HNPP at the age of 30s. It is possible that father may have a late onset of HNPP. There is no report indicating that seizure or IS is a feature of individuals with HNPP [33]. Therefore, it is less likely that *PMP22* related CNV found in proband is a risk factor for IS and additional investigation is warranted to search for another possible cause in this case.

### Probably benign CNVs

Five CNVs in cases of S2, S34, S42, S75, and S133 are probably benign or non-pathogenic to IS despite their rare occurrence in the controls. The sizes of these CNVs were small. There are no functionally significant genes within these CNVs. The inheritance is either *de novo*, inherited, or unknown. We thus reasoned that these CNVs are more likely to be benign or very unlikely to contribute to IS.

## Discussion

Here, we report the first genome copy number variation analysis in a cohort of 47 Chinese children with IS. Our study identified four pathogenic CNVs (17p13.3, 1q44, 2q23.1, and 1q21.1) involving genes of *PAFAH1B1*(known), *HNRNPU* (known), *MBD5* (new), and *CHD1L* (new) in 47 Chinese children with a primary diagnosis of IS. The 17q13.3 deletion containing *PAFAH1B1(LIS1)* gene is known to be responsible for IS [16]. The finding of CNVs at 1q44, 2q23.1, and 1q21.1 in IS is novel and reported for the first time. The overall positive detection rate for pathogenic CNV in this cohort was 8.5% (4/47), which is similar or slightly higher than the rates reported in other studies of IS [8,37]. The detection rate of epilepsy syndromes other than infantile spasms or epileptic encephalopathy varied [9,11]. This difference is most likely related to patient selection in different studies. As noted, the patients in our cohort have a higher percentage of congenital anomalies that may contribute to the higher positive detection rate of CNVs in our study.

The identification of a small 115-kb microdeletion disrupting *PAFAH1B1*(*LIS1*) gene is consistent with the clinical presentations of lissencephaly and IS [16]. The CNV at 1q44 containing *HNRNPU* has been reported in cases with seizures, brain malformation, and developmental delay [38,39]. However, an association of this CNV with IS has not been reported previously. More importantly, a sequence in the splice acceptor site of *HNRNPU* in individual with IS was recently reported by WES in epi4K project [4]. Together, these results indicate a pathogenic role of the 1q44 deletion, and primarily, *HNRNPU* deficiency in IS. The CNVs at 1q21.1 and 2q23.1 have been previously implicated in a wide spectrum of clinical presentations including intellectual disabilities, congenital anomalies, ASD, and generalized seizures but not in IS [19,20]. These may simply be due to the clinical information in these reports were typically extracted from limited medical records for the subjects in the studies. In other cases, the features of IS may be age dependent and missed at time of recruitment. Our findings then support a careful review of clinical records or re-evaluation clinically to determine whether IS is more prevalent in 2q23.1 deletion cases. The *MBD5* gene within the 2q23.1 deletion has been shown to be the primary gene responsible for neurological features of ASD, seizures, and intellectual disability [20,22,23]. Recently, a deleterious mutation in *MBD5* was found in a patient with epileptic encephalopathy [40]. The *CHD1L* gene in 1q21.1 is interesting candidate. The known functions of *CHD1L* in the literature are primarily related to cancers [26]. However, it was noted that CHD2, a protein that belongs to the same family as CHD1L, has been strongly implicated in epileptic encephalopathy in several recent studies [40,41]. Together with our study, these results support a conclusion that *MBD5* and *HNRNPU* play an important role in the susceptibility to IS and epileptic encephalopathy. Our study is the first to report these pathogenic CNV in Chinese children. These findings then suggest that, despite the different genome architecture of CNVs between Caucasian and Chinese populations [13-15], the pathogenic CNVs appear conserved between two populations.

The clinical presentations of our case with a deletion at 2q23.1 shares similar feature but has distinct and more severe neurological presentations than other cases with similar sized deletions [20,22-24] (Table 2). These may suggest additional genomic variants in genome may modify clinical phenotypes. The hypotonia appeared to be more prominent and the onset of seizure was much earlier than in other cases reported in literature. Interestingly, while mild finger anomalies are common in patients with 2q23.1 deletions, the absence of the great toes has not been reported previously. The physical sign of absence of the great toes is not suggestive of other causes, such as amniotic band sequence, because the second toes are apparently larger than expected (Figure 3D). The absence of the great toes is a rare clinical manifestation in literature. The interesting question is whether this is an expanded clinical feature of the 2q23.1 deletion syndrome or whether it has a different cause other than the 2q23.1 deletion.

The combination of keyword searching using ‘seizure’ and ‘absent great toes’ in the London dysmorphology database, a comprehensive dysmorphology database for genetic syndromes, revealed no entries [42]. A search of PubMed using the key words ‘absent great toes’ revealed only one case report of hypoplasia of hallux that was associated with microphthalmia, facial anomalies, microcephaly, thumb hypoplasia, and agammaglobulinemia [43]. These presentations are clearly different from our case. It remains to be seen whether the feature of absent great toes is unique to the Chinese children with *MBD5* related 2q23.1 deletions. If this is caused by a different genetic cause, we speculate that the unique presentation of microcephaly, infantile spasms, and absent hallux may define a new genetic syndrome requiring further delineation in additional cases.

To our knowledge, there are three genome-wide CNV studies specifically to IS in the literature [7,8,37]. Two similar studies in Caucasian children reported previously [8,37]. Another report analyzed CNVs using data from cases with IS that were referred to a clinical diagnostic laboratory [7] (Table 3). A large WES study on IS was reported as part of the epi4K project on epileptic encephalopathy [4,18]. Using data from a CMA performed in a clinical genetics laboratory, Paciokowski et al. reported 11 CNVs in 11 patients referred for a clinical genetics evaluation of IS [7]. Mefford et al. included 44 infantile spasms cases in a large-scale CNV analysis for 315 children with epileptic encephalopathy. Three pathogenic CNVs were identified in 44 cases of IS (3/44 = 6.9%) [37]. These include a *de novo* deletion in *CDKL5*, an inherited duplication of 16p11.2, and an inherited deletion of 12q12. In the last study, Tiwari et al. identified 4 known CNVs in 11 IS cases. These include a *de novo* duplication of maternal origin in the 15q11-q13 region, a deletion of Xp22.13 containing *CDKL5*, a deletion of 16p11.2 and an inherited deletion of 2q32.2 [8]. Although the sample size was still small, our study provides further evidence from a different population supporting the role of CNVs in the etiology of IS. More importantly, the identification of CNVs in 1q44 and deletion of 2q23.1 indicate that *HNRNPU* and *MBD5* genes play significant roles contributing to the susceptibility of IS.

**Table 3:** Comparison between this study and published CNV and WES studies of infantile spasms

**CNV studies**					**WES (epi4K)**^**4,18**^
**No of case**	**This study**	**Mefford, et al**[37]	**Tiwari, et al**[8]	**Paciokowski, et al**[7]	**Epi4K Consortium & Epilepsy Phenome/Genome Project**
	47	44	13	N/A (clinical case series from diagnostic lab)	infantile spasms ( n = 149) Lennox–Gastaut syndrome (n = 115).
**Pathogenic/Possible pathogenic CNV**	**4**	**3**	**4**	**11**	329 de novo mutation
	**De novo:**	**De novo:**	**De novo:**	**De novo:**	**Mutations in more than one proband**
	17p13 115 kb del	Xp22 290 kb del	15q11 4.8 Mb dup	2p16.1 4.0 Mb del	SCN1A; STXBP1; GABRB3;
	1q44 2.1 Mb del	16p11. 2.5 Mb dup	16p11.2 595 kb del^a^	3p26.3 1.6 Mb dup	CDKL5; SCN8A; SCN2A;
	2q23.1 4.1 Mb del	3q11 1.64 Mb dup	Xp22.11 del^a,b^	7q11.23 1.4 Mb del	ALG13; DNM1; HDAC4
	1q21 2.1 Mb dup			14q12 3.3 Mb dup	
			**Inherited:**	14q32.3 317 kb dup	**Other candidate genes:**
			2q32.3 421 kb del	15q11-q13 5.3 Mb dup	CACN1A; CHD2; FLNA;
				19q12 1.3 Mb del	GABRB1; GRIN1; GRIN2B;
				20p13 1.9 Mb del	HNRNPU; IQSEC2; MTOR;
				21q21 1.3 Mb dup	NEDD4L
				21q21 1.3 Mb dup	
				Xp22.2 435 kb dup	
				**Unknown**	
				2q24.3 21.5 Mb dup	

^a^The two CNV observed in one proband.

^b^Size is unknown.

del, deletion; dup, duplication.

*MBD5* belongs to the methyl-CpG DNA binding protein family that includes *MeCP2,* a gene that causes Rett syndrome [44]. *MBD5* is highly expressed in the brain but its function in brain remains uncharacterized [45]. Mice with null mutations in *Mbd5* displayed growth retardation, small brain size, and early postnatal lethality [45]. These results indicate that *Mbd5* is important for brain development but the exact role of *Mbd5* remains to be defined. Because of the haploinsufficiency of *MBD5* in humans, an interesting question for investigation is whether the *Mbd5* heterozygous mutant mice recapitulate the clinical features observed in humans, including IS. *HNRNPU* belongs to the family of heterogeneous nuclear ribonucleoproteins (hnRNPs) proteins that associate with nascent RNA polymerase II transcripts to form hnRNP complexes [46]. *HNRNPU* is thought to influence the structure of hnRNA and to participate in pre-mRNA processing but the function of *HNRNPU* in the brain has not been studied.

Generalized seizures have been recognized as one of the common comorbidities in ASD [47]. The clinical association between ASD and IS is also well recognized [2]. However, the exact molecular link between ASD and seizure remains poorly understood. The topic as to whether seizure or autism is the primary pathophysiology is a subject for ongoing debate. In cases with both IS and ASD, chronologically, the infantile spasms usually present before the signs and symptoms of autistic features emerge, thus the pathophysiology of IS may be the primary mechanism for ASD. However, it is equally possible that different pathophysiology at different developmental stages is responsible for IS and ASD. Together with other genes such as *TSC1*/*2* and *CDKL5*, the implication of *MBD5*, a strong ASD causative gene, in IS supports the molecular link between ASD and IS. Our findings certainly support a medical re-sequencing study of *MBD5* in a large number of children with IS to consolidate the role of *MBD5* in IS, and provide an opportunity to further dissect the molecular link between the IS and ASD.

## Conclusions

We reported the first genome wide copy number variation analysis in Chinese children with IS. Our findings of the CNVs of 1q21.1 gain and 1q44, 2q31.1, 17p13.3 loss in Chinese IS children not only support the pathogenic CNVs are conserved across different ethnic backgrounds, but also specifically implicate the *MBD5* and *HNRNPU* genes in IS. The finding of *MBD5* in both ASD and IS also offers the opportunity to dissect the shared molecular pathophysiology between ASD and infantile spasms.

## Abbreviations

IS: Infantile spasms; ASD: Autism spectrum disorder; CNV: Copy number variation; CGH: Comparative genomic hybridization.

## Competing interests

All authors declare no conflict interest.

## Authors' contributions

XD, YA, LY, RL, YQ, XG conducted the experiments and data analysis. XD, LY, SZ and DS contributed to the patient recruitments. BLW contributed to the array CGH analysis. DX, YA, YHJ and YW designed the experiments, data analysis, and wrote the manuscript. All authors read and approved the final manuscript.

## Authors’ information

Ms. Xiaonan Du, the first author of paper, is the PhD student of Children’s Hospital of Fudan University. This study is part of her dissertation work. Dr. Yi Wang, the corresponding author of the manuscript, is Professor and Division Chief of Neurology, Vice President of Children’s Hospital of Fudan University. Dr. Bai-lin WU is Assistant Professor and the Director of Laboratory of Medicine at Boston Children’s Hospital, Harvard School of Medicine. Dr. Yong-hui Jiang is Associate Professor of Pediatrics and Neurobiology at Duke University School of Medicine.

## Pre-publication history

The pre-publication history for this paper can be accessed here:

http://www.biomedcentral.com/1471-2350/15/62/prepub

## Supplementary Material

Additional file 1: Table S1 Clinical characteristics of studying subjects in this study. **Table S2.** The primers for validation of rare CNVs by real-time PCR. **Table S3.** The primers for Sanger sequencing of MBD5 and ORC4 genes. Click here for file
