# Supplementary material for: A genomic copy number variant analysis implicates the MBD5 and HNRNPU genes in Chinese children with infantile spasms and expands the clinical spectrum of 2q23.1 deletion
Source: BMC Med Genet. 2014 May 29;15:62. doi: 10.1186/1471-2350-15-62 (PMC4061518; doi:10.1186/1471-2350-15-62)
Supplement: Additional file 1: Table S1 — Clinical characteristics of studying subjects in this study. Table S2. The primers for validation of rare CNVs by real-time PCR. Table S3. The primers for Sanger sequencing of MBD5 and ORC4 genes. [file 1471-2350-15-62-S1.docx]

**Supplementary Information**

**Supplementary Table S1. Clinical characteristics of studying subjects in this study**

| Subject | Sex | Spasm ,On-set age (Month) | EEG (HYPS+/-) | Brain MRI | Development prior to seizure onset | Development at Last Review | Other Clinical features |
| --- | --- | --- | --- | --- | --- | --- | --- |
| **Subject with brain malformation** | | | | | |  |  |
| S15 | F | 2 | + | Microcephaly(<3SD),  diffuse pachygyria | Global Delayed | Severe delayed | Horizontal eye nystagmus, Hypotonia |
| S100 | M | 4 | + | Microcephaly(<2.5SD),  diffuse pachygyria, possible Lissencephaly | Delayed | Severe delayed | Hypotonia, [recurrent](app:ds:recurrent) [respiratory](app:ds:respiratory) [tract](app:ds:tract) [infection](app:ds:infection), feeding difficulties |
| S111 | F | 2 | + | Heterotopia at lateral ventricle ，thin corpus callosum, diffuse pachygyria | Delayed | Severe delayed | Hypotonia |
| S122 | F | 2 | + | Thin corpus callosum, diffuse abnormal migrational pattern , possible polymicrogyria | Delayed | Severe delayed | Hypotonia, [visual](app:ds:visual) [impairment](app:ds:impairment) |
| S165 | M | 6 | + | Suspected Dandy-Walker malformation，loss of right corpus callosum ，heterotopia | Mild Delayed | Severe delayed | Hypotonia, |
| **Subject with other congenital abnormalities** | | | | | |  |  |
| S1 | M | 6 | + | Normal at 1.5 months and cortical dysplasia at 1 years old | Mild Delayed | Global delayed | Microcephaly, congenital malformation of auricula |
| S35 | M | 6 | + | Right frontal and parietal lobes are smaller than left, abnormal signal in the cerebellum | Mild Delayed | Global delayed | congenital heart disease (ventricular septal defect and atrial septal defect) |
| S37 | M | 5 | + | delayed myelin development and cortical dysplasia | Mild Delayed | Global delayed | autism feature, loss of language |
| S76 | F | 4.5 | + | discreased white matter | Normal | Global delayed | Hexadactyly of little finger |
| S107 | F | 7 | + | Prominent ventricles | Severe delayed | Severe delayed | Microcephaly, congenital vertical talus(loss of correction) ,visual developmental disorder(corticl blindness) and feeding difficulties, disproportion of the body(short arms) |
| S162 | F | 6 | + | suspected cortical dysplasia | Global delayed | Profound delayed, and no verbal at 1 years old | Microcephaly, Loss of the big toe, Hypotonia especially the [lower](app:ds:lower) [limbs](app:ds:limbs), loss of eye contact and autism features. Short fifth digits of hand, loss of language |
| S168 | M | 5 | + | normal | Mild delayed | Global delayed | Micropenis, obesity |
| **The other subjects** | | | | | | | |
| S2 | M | 3 | + | Prominent extracerebral cerebrospinal fluid space | Normal | Mild delayed | Seizure controlled from 1.5 years old |
| S3 | F | 6m | - | Frontal gyrus dysplasia | Global delayed | Severe delayed | Ohtahara syndrome at 1months old, and spasms at 6 month |
| S4 | M | 4 | + | Mild extracerebral cerebrospinal fluid space | Normal | Severe delayed |  |
| S7 | M | 6 | + | delayed myelin development | Normal | Global delayed | Feeding difficulties, Microcephaly |
| S9 | F | 4 | + | normal | Normal | Global delayed |  |
| S11 | F | 8 | + | abnormal myelin development, enlarged left ventrile | Normal | Global delayed |  |
| S14 | M | 7 | + | normal | Normal | Global delayed |  |
| S23 | M | 6 | + | normal | Mild Delayed | Profound delayed, unable to walk at 2 years old |  |
| S33 | F | 6 | + | normal | UK | Global delayed |  |
| S34 | M | 5 | + | normal | Normal | Global delayed |  |
| S42 | M | 4 | + | normal | Normal | UK |  |
| S60 | M | 6 | + | Mild extracerebral cerebrospinal fluid space | Normal | Severe delayed, no verbal at 5 years old |  |
| S67 | F | 10 | + | Mild extracerebral cerebrospinal fluid space | Mild Delayed | Severe intelligent disability |  |
| S75 | M | 6 | + | Normal | Normal | Mild -Normal |  |
| S86 | F | 4 | + | Normal | Normal | Global delayed, unable to walk at 2 years old |  |
| S89 | F | 4 | + | normal | Normal | Global delayed |  |
| S93 | F | 6 | + | Mild extracerebral cerebrospinal fluid space | Normal | Profound delayed | Transformed to LGS at 2 years old |
| S102 | M | 12 | + | suspected hyperplasia of Right frontal lobe | Normal | Global delayed |  |
| S109 | F | 9 | + | normal | Normal | Global delayed | Transformed to LGS at 2.8 years old |
| S110 | M | 6 | + | smaller brain-stem ,Mild cerebral white matter dysplasia | Normal | No language and autism features | Transformed to LGS at 4 years old and diagnosed with ASD at 5 years old |
| S121 | F | 2 | + | normal | Normal | Global delayed |  |
| S125 | F | 5 | + | Mild extracerebral cerebrospinal fluid space | Normal | Mild delayed |  |
| S129 | F | 7 | + | Mild extracerebral cerebrospinal fluid space | Normal | Delayed , no verbal at 2 years old |  |
| S131 | M | 5 | + | normal | Normal | Profound delayed | Transformed to LGS at 2.5 years old |
| S133 | M | 13 | - | normal | Delayed | Global delayed |  |
| S133B | M |  |  |  | Delayed | Severe delayed , unable to walk at 7 years old | The sibling with S133 |
| S134 | F | 4 | + | decreased white matter | Normal | Global delayed |  |
| S135 | F | 4.5 | + | enlarged left ventricle | Normal | Delayed |  |
| S137 | M | 6 | + | normal | Mild delayed | Global delayed |  |
| S159 | F | 5.5 | + | Mild extracerebral cerebrospinal fluid space | Delayed | Profound delayed | Transformed to LGS at 2 years old |
| S161 | F | 4 | + | normal | Normal | Global delayed |  |
| S163 | F | 4 | + | normal | Mild delayed | Delayed, only can sit at 1 years old |  |
| S171 | M | 6 | + | normal | Normal | Delayed, unable to sit alone at 11mo |  |
| S173 | M | 8 | + | normal | Normal | Delayed , unable to walk at two years old, No language | Diagnosed with ASD at 4 years old. |

**Supplementary Table S2: The primers for validation of rare CNVs by real-time PCR**

| **Case** | **CNVs region** | **Genomic region** | **Key genes** | **Primer name** | **Forward primer** | **Reverse primer** |
| --- | --- | --- | --- | --- | --- | --- |
| S2 | dup | chr:216898976-217160487 | XRCC5 | XRCC5-1 | GATCTTCCCCCTTCCTATGC | GAGACACCCCCAGAGTTTCA |
|  |  |  |  | XRCC5-2 | TTAGGACTGGCCAATTACCG | GGCACAGTTGAATGCTGTTG |
|  |  |  |  | XRCC5-3 | TCAACCCACACAAACTGCAT | GTGTTTCATGGTGTCGGCTA |
|  |  |  |  | XRCC5-4 | AACCTGCTTGAGCTGCCTTA | TGCCTCCCCAAGATACTCTG |
|  |  |  |  | XRCC5-5 | TGTGCTTTCACTCACCTTGC | TCGGCTTTCTAATTGGTTGG |
| S4 | del2p22.1 | chr2:39,328,883-39,336,541 | SOS1 | F1/R1 | TCCATCAAACCCAAGACCAG | TGCACTTAGAATTTTTGCACCT |
|  |  |  |  | F2/R2 | ATTGTGCTCGCATAGTCGTG | CCCCAGGCTTTGATAACTGA |
| S15 | dup10q23.1 | chr10:84,222,075-85,293,140 | NRG3 | F1/R1 | TTCAAAGGTGGAAAGGCATC | AAGTCATGCTGCCAGTCCTT |
|  |  |  |  | F2/R2 | ATCAGGATGGATGGGAATGA | GGCCATCCCTGAATGAAGTA |
|  |  |  |  | F3/R3 | AGCATCAAATGGTGCAAAAA | GTTTCTACGCTGGCCAGTTC |
| S34 | dup | chrX:102262951-102659333 | NGFRAP1 | NGFRAP1-3 | TCTCGGTCCTCTCCAATCTG | ACACGCAAGGGGTAGAAGTG |
|  |  |  |  | NGFRAP1-4 | CGGTCTTCCTCTCCATTCTG | GTCAGAGCACCAAGCATTCA |
|  |  |  |  | NGFRAP1-5 | GCAACACAAGACCCACTTGA | GGGGGAGCTCTCTAATCACC |
| S34 | dup2q22.1 | chr2:137,826,656-138,048,583 | THSD7B | F1/R1 | GCCATGTCCTCATGGAATCT | CGGCCTTCAGAATACGATGT |
|  |  |  |  | F2/R2 | AGGAGGGGTTTTCTTCTCCA | CACACACACCCCTTATGCAG |
|  |  |  |  | F3/R3 | TGAGAGTTGCCACAGACAGG | TGCCCATGAGAGAAACACAA |
| S42 | dup4p11 | chr4:48,983,002-49,063,489 | CWH43 | F1/R1 | CCAGTGGGCTCTCATGATCT | ACTTGCCAAGCACAGCAGTA |
|  |  |  |  | F2/R2 | CAGCTTCCCTTGCATCTAGC | AGATCCCAAGATTGCTGTGC |
|  |  |  |  | F3/R3 | ATGCAAATGTGTTGCCAGAG | AAAAGGGATGTGTCCCACTG |
| S37 | del1q44 | chr1:244,961,797-247,074,490 | SMYD3 | F1/R1 | ACTGTGCAAACCAGTGCAAG | TGGGTGATCATTTCAGCAAA |
|  |  |  |  | F2/R2 | CCTTGAGTGAAAGGGCTCAG | TTGTTCTCAGGGGGACTTTG |
|  |  |  |  | F3/R3 | TCTTCATTGCTTGGGGAAAC | TGAAATGCTGACTGCAAAGG |
| S67 | dupXp22.3 | chrX:463,789-877,737 | SHOX | F1/R1 | TTGGCAGCTCTTCCTCAAAT | ATACTCGCTGCTTTCCCAAA |
|  |  |  |  | F2/R2 | TATAGGGGCAACACATGCAA | GAGGACACCCCTCTCCTTTC |
|  |  |  |  | F3/R3 | AGTGCCGCAAACAAGAGAAT | TACCTGTTGGAAAGGCATCC |
| S67 | dup1q21.1 | chr1:145764453-147824207 | CHD1L | CHD1L-1 | TCCGAAGAGCAGACATTTCA | ACAGGCTACTTTGGGGCTTT |
|  |  |  |  | CHD1L-3 | ATGACTGGGACACCTGGAAG | TCCCAAGAATATGGGATGGA |
|  |  |  |  | CHD1L-4 | CAGAGGCCAGCAGTTTATCC | TAAATGGCCCTGGCTGTTAC |
| S75 | dup6q22.31 | chr6:1249111-118989529 | FAM184A | F1/R1 | AATGTTGCTTCCTGCACCTC | GCCACAGTGTCAGTCAGTGC |
|  |  |  |  | F2/R2 | CATGACCCAATCAGGAAAGG | TGGTTTGCCTGCTAGCTGAT |
|  |  |  |  | F4/R4 | GGTGGAGCCCAAGACAAATA | TATGAAGGCGAAAATGCACA |
| S100 | del17p13.3 | chr17:2,405,454-2,520,464 | PAFAH1B1 | F1-N/R1-N | CCTCCTCCCTCGGTGACTTA | CGATAACTGAGGGTGGGGTG |
|  |  |  |  | F2-N/R2-N | CCGTTTCGAGACCTGGAGAG | CTTGGGAGGAGGAGCTGTTG |
|  |  |  |  | F3/R3 | TCGAATTCGCGAGAAAACTT | TGAGTGAGACATCGGCTGAG |
| S134 | del17p12 | chr17:14,111,754-15,442,119 | PMP22 | F1/R1 | CTGGAAGCAAGGAAGGTGAG | CCAGGCGTATCAACAGGAAT |
|  |  |  |  | F2/R2 | ACCCTTGCCCTGTAAGGTCT | GATTTGGTGGGGAGAGTGAA |
|  |  |  |  | F3/R3 | TGGACCGAAGGGAGTAGATG | CAGCCATGCATGTCCTCTAA |
| S133 | dup8q24.22 | chr8:135,644,952-135,791,363 | ZFAT | F2/R2 | GCATCTCTAGCCAACCAAGG | CAGCATCCCTCCTACTCTGC |
|  |  |  |  | F3/R3 | TAACCCCTTCTTCCATGTGC | AGAAGGGCAGCTTTCGGTAT |
|  |  |  |  | F4/R4 | CCGCATCTCTAGCCAACCAA | GTGTAGATGGAGAGGCGGTG |
| S159 | dupXq26.2 | chrX:133,593,799-133,594,594 | HPRT1 | F7/R7 | GGTAGCCGTGGGAATTTTCT | AGTGCAAGGTCTTGGGAATG |
| S162 | del2q22.3 - q23.3 | chr2:147953313-152061251 | MBD5 | F1/R1 | ACTTTTGAAGGCCATCATGC | GCTGGAAGACCTCCTTCCTT |
|  |  |  |  | F2/R2 | GGGTCTTTGTTATCTTGCTTGG | TGAAATGGTTACCTTGGGAAG |
|  |  |  |  | F3/R3 | GGCTGTGCTTTTTCCAGGTA | GGGCTTCCATGCTTTTATGA |
|  |  |  |  | F4R4 | TCTACCCCCGACAGAGATTG | GAATTGGGAGCTCCATGAAA |
|  |  |  |  | F5/R5 | TTGCGGAAAAGAAAACAACC | CACAACCCGGGGTACTATTG |
|  |  |  |  | F6/R6 | TTCCATCCAACAGCACTTCA | CACAGCGTGGACAGAATCCAG |
|  |  |  |  | F7/R7 | GCTTTCCTCGTGGAATTGG | TGGAGAAGTTGCTGCTGTTG |
|  |  |  |  | F8/R8 | AGCACTGCAAAGCAAGACCT | CCATTAACGCTTTTGGCATT |
|  |  |  |  | F9/R9 | AGCCCGAGAAGTTGAAGACA | CACAGCGTGGACAGAATCAG |
|  |  |  |  | F10/R10 | GCTTTCTCTCGTGAATTGG |  |
|  |  |  |  | F11/R11 | ACTGACATCTGGGGTCTGG | ACCCTGTGGGATTTGGTGTA |
| S163 | dupXp22.3 | chrX:6,451,691-8,115,193 | HDHD1 | F1/R1 | TTCCATAAAAGGCCATCTCG | GGTGGCCTTTGTTGTCAGAT |
|  |  |  |  | F2/R2 | GCTTGTGCCAAGAGGTTCTC | GCCACGATTCCTCACAAAAT |
|  |  |  |  | F3/R3 | CTGCAAGCCTTTCTCCTGTT | CCACTGAATACAGCCGTTCA |
| S165 | del11q22.1 - q22.2 | chr11:101857720-102256635 | YAP1 | YAP1-1 | GTCATCGCTTCCCAAACATT | CCGGCTTGCTCTTATCAAAC |
|  |  |  |  | YAP1-3 | TGAAACCCAGGATTTGTGGT | TCACCCAGCAGCACTGTAAG |
|  |  |  |  | YAP1-5 | GATCCTCTGGCTCCGAATTT | CCTGGTTGTGAAGGCATCTT |

**Supplementary Table S3: The primers for Sanger sequencing of MBD5 and ORC4 genes**

| **MBD5** | | | |
| --- | --- | --- | --- |
| **Primer Name Forward Primers** | | **Reverse Primers** | |
| MBD5_E7_F | TGCCTGCTTCTGTCCTTTCT | MBD5_E7_R | GCATTCCAAAACACATGCAA |
| MBD5_E8_F | TTTGCCACTAAAAGCTTGAGTTC | MBD5_E8_R | TGTGCGTGCCTGAAAAGTAG |
| MBD5_E9-1_F | CCTCTCCCTTCCTGATTTCC | MBD5_E9-1_R | AGCCATTGCACAGGATGACT |
| MBD5_E9-2_F | TTTCATGGAGCTCCCAATTC | MBD5_E9-2_R | TGCCTCAATCCTTCCAATTC |
| MBD5_E9-3_F | TCCCCTTCTCCAGTGACATC | MBD5_E9-3_R | TGGCTGCTGCTGAGAGTAAA |
| MBD5_E9-4_F | CCATCAAGCCCTTCTACCAA | MBD5_E9-4_R | CACAACCCGGGGTACTATTG |
| MBD5_E9-5_F | TTGCGGAAAAGAAAACAACC | MBD5_E9-5_R | ATTTCACAATGGGGAAAGGA |
| MBD5-E9-6F | GAACTCCACCCTGTCTACCC | MBD5-E9-6R | AGGGTCAAGAATGCCAAGTG |
| MBD5-E9-7F | CGAGCAATGTTCCACCACAA | MBD5-E9-7R | TGTTGACCTTGGTGACCTGG |
| MBD5-E9-8F | CCACTTGGCATTCTTGACCC | MBD5-E9-8R | AGTAGGAAAAGCTGCACTGC |
| MBD5_E10_F | AAGGTGCCATGGAACAAATAA | MBD5_E10_R | CCTTAAGCAAGCTGTGACATA |
| MBD5-E11F | ACCCAAGTAGCATAGCACTT | MBD5-E11R | AGGGCTTATGAGGGTCACAC |
| MBD5_E12_1F | AATGTTTCGCAGTGATGATTT | MBD5_E12_1R | CCCCTTGTTGATACGACATTT |
| MBD5_E12_2F | AATGTTTCGCAGTGATGATTT | MBD5_E12_2R | CCCCTTGTTGATACGACATTT |
| MBD5_E12_3F | AATGTTTCGCAGTGATGATTT | MBD5_E12_3R | AATTCCTCGTACTTCCACCTG |
| MBD5-E12-4F | GTCCTTGTCATGAAAGGCCC | MBD5-E12-4R | CGGGGCTTTGTTGACATGAA |
| MBD5_E13_F | GGAGCCACAGAATCAACCTC | MBD5_E13_R | GAATGAAGATGCAGGGAATGA |
| MBD5_E14_F | GCTTTCTCTCGTGGAATTGG | MBD5_E14_R | TCAAGTGCAGAAAGCTGTGG |
| MBD5_E15_1F | CTGACATCTGGGGTCTTGGT | MBD5_E15_1R | ACACCCGAAATTGAGTCCAG |
| MBD5-E15-2F | TCCACTGTCTATTGGCACCC | MBD5-E15-2R | TGTGGTGGTGGTAGCTATCTG |
| **ORC4** | | | |
| ORC4-Exon 02F | TGAAGCCATCGAAGATTTTG | ORC4-Exon 02R | TGTGCTGGCATCTCGTTAAG |
| ORC4-Exon 03F | AATTTAGGGAGGGTTGAATTG | ORC4-Exon 03R | CAAAACCTGGAAGGCATCTG |
| ORC4-Exon 04F | GCTAGGCCAAGTACCCAGAG | ORC4-Exon 04R | CACTAGGAGGACAAATAAGGGG |
| ORC4-Exon 05F | AATTTGAAAACTTACTTGTTAAAGAG | ORC4-Exon 05R | TGGTGTGCTAAGTAAAACATGG |

| ORC4-Exon 06F | AACTGGAAAACAACCTGCTG | ORC4-Exon 06R | GAATTTCTGAACCTCTCCCTACC |
| --- | --- | --- | --- |
| ORC4-Exon 07F | TTTGAGAATCAGACAGCCATC | ORC4-Exon 07R | TTTGGCAAAAGCTTGTATTACC |
| ORC4-Exon 08F | CCAAAGTAGGCTATCAGAATGTTTAC | ORC4-Exon 08R | AACTAGCAGTGTCAGCAATTAAGC |
| ORC4-Exon 09F | CATTGGTACAGTGTTTTTGTTTTT | ORC4-Exon 09R | GCAGCATTATCCTTCAGCAA |
| ORC4-Exon 10F | ACCAACCAGTAAGGCACAGG | ORC4-Exon 10R | AGTTCGCAACCAGTCTGAGC |
| ORC4-Exon 11F | TCAGAAGTTTTGCACAGTATCTCC | ORC4-Exon 11R | GCATTATGCCCACGTTAATTG |
| ORC4-Exon  12-13F | TGACCTTCTTCACCCCTCAG | ORC4-Exon 12-13R | GGGCAGTATACCTCCAC A A AC |
| ORC4-Exon 14F | GCTTCCCAGTACTCTGTTCTGC | ORC4-Exon 14R | GGACAATAGTTTTCCGTTCTCTAC |
